# Gay and Bisexual Men Living with Prostate Cancer: From Diagnosis to Recovery

**DOI:** 10.5195/jmla.2019.767

**Published:** 2019-10-01

**Authors:** Gregg A. Stevens

**Affiliations:** Health Sciences Library, Stony Brook University, Stony Brook, NY, gregg.stevens@stonybrook.edu

As stated in this book’s introduction, it is estimated that approximately 2.8 million people in the United States are living with prostate cancer: living with a diagnosis, undergoing treatment, or living in the posttreatment recovery stage. Using the estimate that about 4% of men in the United States are gay or bisexual, this means that about 100,000 gay or bisexual men have been diagnosed with prostate cancer. Another statistic cited in the introduction is the fact that 1 out of 6 gay and bisexual men in the United States will be diagnosed with prostate cancer sometime in their lifetime (p. 3).

As a cancer that is tied so tightly to sexuality, it would be reasonable to assume that prostate cancer patient care and information would encompass a variety of sexual preferences and practices, as a way of being culturally competent and addressing patient needs. However, as this volume of essays and research studies shows, a major disparity exists in the quality of care and information received by gay and bisexual men who are living with prostate cancer.

The book’s chapters are grouped in three broad sections. The first section provides an overview of the current research base regarding the experiences of gay and bisexual men with prostate cancer, both physically and emotionally. As described in several chapters throughout this collection, prostate cancer is a disease that affects not only the patient, but also the patient’s partner. Hetero-centric patient support has focused on the wife’s role in the patient’s care and recovery as the primary emotional support and sexual partner. Gay and bisexual men living with prostate cancer have different types of relationships to consider.

Most of these early chapters discuss sexual identity and the patient’s place in a same-sex relationship or in the patient’s social network of friends and family. This section focuses on the importance of these nonbiologically based social networks and relationships in the treatment of and recovery from prostate cancer.

The chapters in the second section deal with cancer care and support for this population during treatment and recovery. Some of the topics discussed include cancer treatment decision making, the effects of radiation therapy on sexual health, and experiences with sexual aids and other forms of sexual rehabilitation after treatment. The final chapter of this section notably discusses the development of Malecare, a gay, bisexual, and transgender prostate cancer support group.

Because sexual practices and interests for gay and bisexual men will differ from those of heterosexual men, it is important to provide relevant advice and information to the patients and the people in their support networks. However, as described across a number of the chapters in the book, many gay and bisexual men do not come out to their health care providers. Many who do are faced with confused or even hostile reactions from physicians and others. For treatment and recovery to be fully successful, culturally appropriate therapies are necessary, and these chapters discuss effective, relevant treatments for gay and bisexual men living with prostate cancer.

The final section, and perhaps the most accessible, provides firsthand accounts from gay and bisexual men of their own experiences with prostate cancer. These personal stories put the research findings from the other chapters into context. The authors recount their experiences in health care systems where providers often do not ask about sexual orientation and make assumptions that might not be appropriate for their patients. Several of these authors describe their proactive efforts to receive care that is responsive to their needs. However, the overall tone of these essays was one of optimism, as more providers become aware of the diverse needs of their patients.

While gay and bisexual men are the primary subjects of the book, some chapters carefully redefine the subject base to include all non-heterosexual persons with prostate cancer. This redefinition allows discussion of transgender women, a small and often neglected part of prostate cancer demographics. Because their numbers are very small, the evidence base is lacking regarding their prognoses and experiences. However, there are enough instances of transgender women with prostate cancer to mention them and their needs, an important inclusion by the authors.

A twelve-page glossary at the end defines many words and phrases used throughout the book, both those of a medical nature and those specific to LGBTQ+ culture and sexuality.

Because of the multidisciplinary nature of this volume, it could be a useful addition to several types of health sciences library collections. It is certainly recommended for libraries seeking to build their LGBTQ+ health holdings, as well as libraries with strong collections in oncology or sexual health. It is also recommended for libraries serving psychology, counseling, or social work programs. While the overall tone of the book is somewhat academic, the firsthand accounts in the final chapters might also make it appropriate for consumer health collections in hospitals.

***Gregg A. Stevens, AHIP****, gregg.stevens@stonybrook.edu, http://orcid.org/0000-0003-4088-6742, Health Sciences Library, Stony Brook University, Stony Brook, NY*

